# Effects of Electroacupuncture on Pain Memory-Related Behaviors and Synchronous Neural Oscillations in the Rostral Anterior Cingulate Cortex in Freely Moving Rats

**DOI:** 10.1155/2019/2057308

**Published:** 2019-05-15

**Authors:** Zui Shen, Yilin Zhu, Boyi Liu, Yi Liang, Qiaoying He, Jing Sun, Zemin Wu, Haiyan Zhang, Shujing Yao, Xiaofen He, Jianqiao Fang, Xiaomei Shao

**Affiliations:** Department of Neurobiology and Acupuncture Research, The Third Clinical College, Zhejiang Chinese Medical University, Key Laboratory of Acupuncture and Neurology of Zhejiang Province, Hangzhou 310053, China

## Abstract

Our previous studies have confirmed that electroacupuncture (EA) can effectively intervene in pain memory, but the neural mechanism involved remains unclear. In this study, we observed the effects of EA in regulating pain memory-related behaviors and synchronous neural oscillations in the rostral anterior cingulate cortex (rACC). During nociceptive behavioral testing, pain memory induced a nonpain stimulus that spurred a neural oscillatory reaction similar to that caused by pain stimuli in the rACC. After EA, nonpain stimuli did not induce decreased neural oscillatory activity in the rACC until the presentation of pain stimuli. During aversive behavioral testing, EA, through the downregulation of theta power, inhibited the retrieval of aversive memory and relieved pain memory-induced aversive behaviors. These changes of oscillatory activity may be the hallmarks of EA therapy for pain memory.

## 1. Introduction

Many studies have proven that initial injury-induced indelible pain memory is often a key factor of long-term unhealed chronic pain [1–4]. Pain memory has been a new focus on chronic pain research [5–7]. Therefore, the eradication of pain memory may be the key to relieving chronic pain. Our previous studies and other studies have shown that the “pain memory” phenomenon can be reproduced in a rat pain memory model, and the present study is also based on this model [2, 3, 8].

The rostral anterior cingulate cortex (rACC) is an important brain area for the modulation of pain memory [2, 3, 9]. Our previous study showed that the cAMP/PKA/CREB signaling pathway in the rACC relating to memory storage and retrieval is involved in regulating pain memory-induced nociceptive behavior in a rat pain memory model [2]. In addition, a previous study indicated that rACC is also involved in the processing of aversive behavior [10]. Thus, whether pain memory induces changes in aversive behavior and rACC is certainly worthy of further study.

Neural oscillation is a rhythmic fluctuation of neuronal excitability. It is a combination of neural ensemble firing with distinct levels of frequency. Delta band oscillation is usually closely related to decision making, attentional process, and expectation [11, 12]. Oscillation in the theta band is related to memory encoding and retrieval [13, 14]. Alpha and beta oscillations are associated with top-down cognitive processes, including attention and preparation of movements [15–17]. Gamma oscillation is commonly observed in many brain regions during both waking and sleep states [18].

Recent studies indicate that the neural oscillation mechanism is central to studying the brain neural network of chronic pain [19–22]. Evidence suggests that neural oscillations in multiple frequency bands modulate different levels of information integration, playing an important role in perceptions, emotions, and behaviors [23–26]. Additionally, pain can induce a change in neural oscillations in brain regions, as recent researches show that acute pain can spur changes in local field potentials (LFPs) in multiple frequency bands in ACC [27–29]. However, whether pain memory induces a change in neural oscillations requires further observation.

Electroacupuncture (EA), a form of acupuncture involving electronic stimulation, is widely applied as an analgesic for chronic pain in clinics [30–32]. EA can regulate the prefrontal neural network which basically overlaps with the neural network of cognition [33]. Additionally, EA can modulate a change in brain oscillatory activity in a rat model of acute pain, regulating the neural network of central processes and spontaneous injury information integration [34]. Our previous research has also indicated that EA can alleviate the retrieval of nociceptive behavior induced by pain memory [2, 3]. However, how EA modulates the neural network in the rat model of pain memory remains unknown and deserves further observation. In this study, to explore the effect of electroacupuncture on pain memory-related synchronous neural oscillations in the rACC in freely moving rats, we observe pain memory-induced nociceptive and aversive behaviors and synchronous LFPs via extracellular multichannel recording in vivo.

## 2. Materials and Methods

### 2.1. Animals and Groups

Male adult Sprague-Dawley (SD) rats of 250-300 g in body weight were obtained from the Experimental Animal Center of Zhejiang Chinese Medical University. The animals were housed in groups of five in plastic cages with soft bedding at the University Animal Care Facility with an artificial 12/12 h light-dark cycle (lights on at 8 a.m.). Animals received food and water ad libitum at a constant room temperature of 23 to 25°C and at a relative humidity of 40% to 70%. Prior to experimental manipulations, rats were given a period of 1 week to adjust to their new surroundings. The whole experiment was performed in accordance with the guidelines of the International Association for the Study of Pain and the Institutional Animal Ethical Committee (IAEC).

Adult male SD rats were randomly divided into a blank control (Control) group, pain memory model (Model) group, and model+electroacupuncture (EA) group.

### 2.2. Surgeries

Animals were anesthetized with urethane anesthesia (1.2 g/kg i.p., Sigma-Aldrich) and fixed to a stereotaxic apparatus (68025, RWD Life Science, China). Rats were placed in a stereotaxic frame on a heated surgical platform maintained at a constant temperature of 37°C. A midline scalp incision was made to expose the skull to allow for the implantation of a microwire array, which was fixed to the skull with surface screws and dental cement. Rats received surgery for neural recordings.

To record the LFPs of rACC neural activity, one recording microwire array was surgically implanted. Surgical procedures used were the same as those used in our previous studies [12]. The array was driven into the right rACC at a twenty-degree angle using a hydraulic microdrive (model 51421, Stoelting Co., Wood Dale, USA). Each microwire array consisted of eight filaments of nickel-chromium wire (35 *μ*m diameter, Stablohm 675; California Fine Wire Company, Grover Beach, CA, USA). The array was constructed in 4 × 2 architecture with 200 *μ*m between the recording wires. The following coordinates (relative to the bregma) were used to center the array: rACC (+2.7 mm rostrocaudal, +0.8 mm mediolateral, and 2.0 mm dorsoventral).

### 2.3. Pain Memory Model

**Figure 1:**
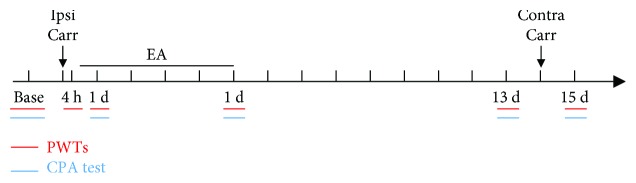
The basic experimental procedure.

In our previous study, the pain memory model was induced by two injections of carrageenan [2, 3]. The first carrageenan injection was placed into the left hind paw plantar surface via the subcutaneous injection of 0.1 mL of 2% carrageenan (Sigma-Aldrich, St. Louis, MO, USA) to induce acute inflammatory pain. After a 14-day recovery period, when the right hind paw was also injected with the same carrageenan, the pain threshold of the recovered left hind paw dropped again. This shows that the pain memory model was successfully prepared. The basic experimental procedure is shown in Figure 1.

### 2.4. Electroacupuncture

Electroacupuncture treatment was applied in rats of the EA group. In a previous study [2, 3], we found that EA treatment at bilateral acupoints “Zusanli” (ST36) and the reference electrode (1 cm inferior of “Zusanli”) were effective for impairing the retrieval of pain memory. Therefore, we used the same methods including the acupoints and EA parameters in this study. The acupoints were needled with stainless acupuncture needles (0.25 mm in diameter × 13 mm in length) and electrically stimulated with a Hans Acupoint Nerve Stimulator (HANS 200E; Huawei Co. Ltd., Beijing, China). The EA parameters were set as follows: 2/100 Hz of the frequency with automatic shifting between 2 Hz and 100 Hz stimulation for 3 s each; a square wave current output (pulse width: 0.2 ms); and an intensity range of about 1-2 mA adjusted to the animals' local muscle contractions. The treatment was administered at 5 h, 1-5 d after the first carrageenan injection. In the whole procedure, all rats maintained relatively comfortable states without any struggling and screaming.

### 2.5. Nociceptive Behavioral Testing

As described above, paw withdrawal thresholds (PWTs) were measured automatically using a dynamic plantar aesthesiometer (model 37450; Ugo Basile, Comerio, Italy) [12, 35]. A paw-flick response was elicited by applying an increasing vertical force (increased steadily from 0 to 50 grams over 20 s) using a stainless steel probe (a straight 0.5 mm diameter shaft) placed underneath the mesh floor and focused at the middle of the plantar surface of the ipsilateral hind paw. According to our previous study, PWT was determined as the mean of four subsequent measurements except for the first at intervals of 1 min [12]. Moreover, all manipulations were made under the guidance of an operator.

### 2.6. Aversive Behavioral Testing

Two carrageenan injections that induced aversive behavior were performed using a modified conditioned place aversion (CPA) paradigm [36]. The plexiglas paradigm consists of two equally sized cabinets (35 × 28 × 45 cm) that can be controlled by opening or closing with a baffle. The two cabinets are composed of wallpaper strips of different widths (3 cm and 9 cm wide) and colors (black and white). One side of the cabinets is 3 cm of alternating black and white colors, and the other side is 9 cm of alternating black and white colors. The base is hollowed out and can be fully placed on the perforated platform which is fitted with a dynamic plantar aesthesiometer.

At baseline, the rats were left free in an open (middle without baffle) paradigm for 30 min, and the activity time of the rats in the two compartments was recorded separately. Pain- and non-pain-paired compartments for each rat were randomly assigned according to the activity time of the rats in the two compartments at baseline. On day 0, the two compartments were separated with the baffle and the rats were placed in a non-pain-paired compartment for free activity for 30 min (unconditioned phase). After the rats were placed back into a cage to rest for 30 min, control rats were given a left hind paw injection of 0.1 mL of 0.9% NaCl and model and EA rats were injected with 0.1 mL of 2% carrageenan in the left hind paw. Four hours later, the rats were placed into pain-paired compartments (conditioning phase), and after a 5 min period of habituation, each left hind paw was stimulated every minute for 25 min using a dynamic plantar aesthesiometer. The rats were placed in the two compartments (middle without a baffle) for free movement for 30 min on day 1, day 5, and day 13, and their activity time in the two compartments was recorded. On day 14, the control rats were given 0.1 mL of 0.9% NaCl in the right hind paw. The model and EA rats were injected with 0.1 mL of 2% carrageenan in the right hind paw. On day 15, the rats moved freely between the two compartments for 30 min, and their activity time was recorded.

Aversive behavior was measured with the CPA score, which is the difference in time between the test day (day 1, day 5, day 13, and day 15) and baseline for the pain-paired compartment. The formula is shown as follows:

(1) $$\begin{array}{c} T_{\text{score}}=T_{\text{test day}}–T_{\text{baseline}}. \end{array}$$

During the experiment, the rats were lightly placed in the central area of the two compartments. After the rats acclimated for 1 min, the video acquisition software automatically started timing and tracking the activity of the rats for 30 min. Real-time recording and data analysis were performed using the SMART video tracking software (v3.0, Panlab, Spain). After the test of the previous rats, the two compartments were thoroughly scrubbed with 10% alcohol to eliminate residual traces and odors that could have affected the activity of the next rats tested.

### 2.7. Neural Recordings

LFPs were recorded from the implanted microwire array with a Cerebus neural signal processing system (Blackrock Microsystems, Salt Lake City, UT, USA). LFP signals were preamplified (300x), bandpass filtered (0.3–250 Hz), and sampled at 1 kHz. Neural recordings were obtained in nociceptive behavioral testing environments in quiet states. A plantar video camera and video tracking system (ANY-maze, Stoelting, CO, USA) was used to generate real-time imaging of the rats' plantar and the stimulus probe in the dynamic plantar aesthesiometer, permitting the synchronization of the probe's stimulus process with the acquired neuronal data. No animals were removed from the study due to the poor placement of recording wires.

### 2.8. Spectral Analysis

Data were processed and validated by offline analysis using NeuroExplorer 5.021 (NEX, Plexon Inc.) and were exported to MATLAB 2014a (MathWorks, Natick, MA) for complementary analysis.

A spectrogram analysis was used to visualize LFP power levels at different frequency bands as a function of time for each condition. The raw rACC LFPs were bandpass filtered at 2-45 Hz using a noncausal zero-phase-shift filter (fourth-order Butterworth). Next, the power spectral densities (PSD) were calculated with Hanning window 2^10^ frequency bins over the 2-45 Hz range and with 50% overlapping windows. The power was normalized by the logarithm of the PSD (in decibels), and smoothing was applied (Gaussian filter, width = 3). The following five frequency band intervals were considered: delta (2–4 Hz), theta (4–9 Hz), alpha (9–15 Hz), beta (15–30 Hz), and gamma (30–45 Hz).

### 2.9. Statistical Analysis

All averaged values are given as the mean ± SEM. One- or two-way repeated measures analyses of variance (rm ANOVA) with Bonferroni post hoc analysis were used when the variances were equal. In all cases, the results were considered to be statistically significant at *p* < 0.05.

## 3. Results

### 3.1. Pain Memory-Related Behaviors Observed during Nociceptive Behavioral Testing

**Figure 2:**
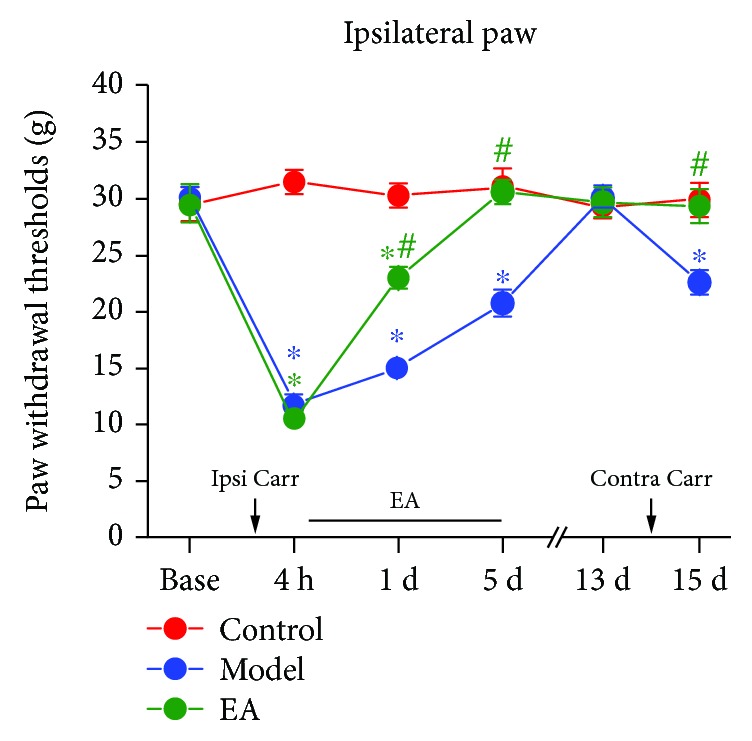
Mechanical withdrawal thresholds of the ipsilateral (left) hind paws in each group. ^∗^*p* < 0.05 compared to the control group and ^#^*p* < 0.05 compared to the model group. *N* = 7. Abbreviation: Ipsi Carr = ipsilateral carrageenan injection, Contra Carr = contralateral carrageenan injection.

As shown in Figure 2, we tested paw withdrawal thresholds (PWTs) of the ipsilateral (left) hind paws in the control, model, and EA groups (two-way rm ANOVA; groups: *F*_(2, 60)_ = 99.973, *p* < 0.001; time: *F*_(5, 60)_ = 62.303, *p* < 0.001; and groups × time: *F*_(10, 60)_ = 18.026, *p* < 0.001; *n* = 7). Post hoc analysis shows that there were no significant differences in each group before the first carrageenan injection was made into the ipsilateral hind paws (*p* > 0.05, Bonferroni test). Furthermore, PWTs in the model and EA groups were significantly decreased compared to those of the control group at 4 h (*p* < 0.05, Bonferroni test). The PWTs of the model group at 1 d and 5 d and the PWTs of the EA group at 1 d were significantly lower than those of the control group at the same time (*p* < 0.05, Bonferroni test), and the PWTs of the EA group at 1 d and 5 d were significantly higher than those of the model group (*p* < 0.05, Bonferroni test). At 13 d, there were no statistically significant differences between each group (*p* > 0.05, Bonferroni test), and at 14 d, the same doses of carrageenan were injected into the contralateral hind paw of the model and EA groups. At 15 d, the PWTs of the ipsilateral hind paw of the model group were significantly decreased relative to those of rats of the control group (*p* < 0.05, Bonferroni test), and compared to those of the model group, the PWTs of ipsilateral hind paws of the EA group were significantly increased (*p* < 0.05, Bonferroni test).

### 3.2. Synchronous Neural Oscillations of Pain Memory-Related Behaviors Observed during Nociceptive Behavioral Testing

**Figure 3:**
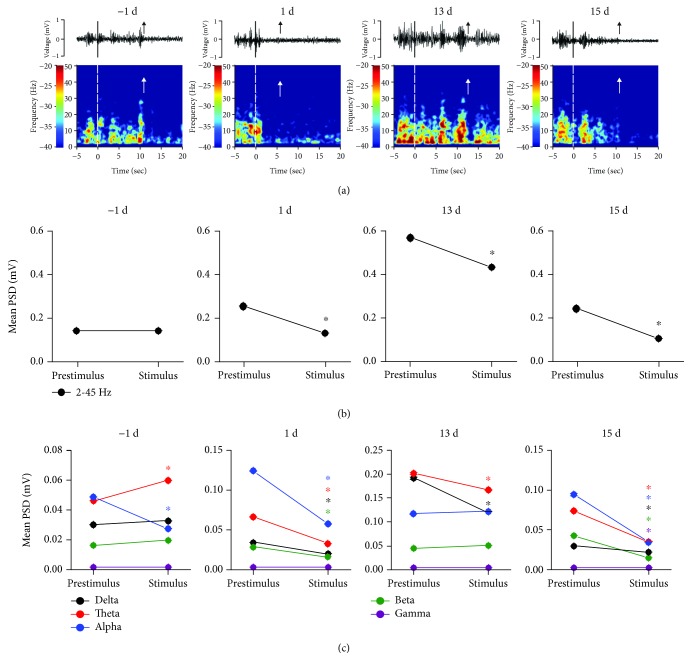
Synchronous LFPs observed during nociceptive behavioral testing. (a) A spontaneous recording and real-time spectrogram for LFP signals of the prestimulus, stimulus, and poststimulus phases (black and white dashed lines indicate the start of the stimulus and the upward arrow represents paw withdrawal). (b) The change in the mean PSD observed within 2-45 Hz between the prestimulus and stimulus phases in the rACC. ^∗^*p* < 0.05 compared to the prestimulus phase. (c) The change in the mean PSD in different frequency bands between the prestimulus and stimulus phases in the rACC. ^∗^*p* < 0.05 compared to the prestimulus phase of the same band. Five frequency band intervals were considered: delta (2–4 Hz), theta (4–9 Hz), alpha (9–15 Hz), beta (15–30 Hz), and gamma (30–45 Hz).

**C**ompared to those of the prestimulus phase (-5 s to 0 s), the amplitude and PSD values observed during the stimulus phase (0 s to paw withdrawal (upward arrow)) showed early downtrends in the rACC at 1 d, 13 d, and 15 d (Figure 3(a)). As the maintenance times of rats in response to the paw withdrawal stimulus varied, we selected mean PSD as the observation target. At -1 d, there was no significant difference in the mean PSD within 2-45 Hz between the prestimulus and stimulus phases (one-way rm ANOVA; *F*_(1, 7)_ = 2,337, *p* > 0.05). At 1 d, 13 d, and 15 d, the mean PSD of the stimulus phase was lower than those of the prestimulus phase (one-way rm ANOVA; *F*_(1, 7)_ = 662.202, *p* < 0.05; *F*_(1, 6)_ = 1114.916, *p* < 0.05; and *F*_(1, 6)_ = 454.370, *p* < 0.05) (Figure 3(b)).

The analysis results of each frequency band are as follows (Figure 3(c)): At -1 d, the mean PSD of the theta frequency band increased during the stimulus phase (one-way rm ANOVA; *F*_(1, 7)_ = 288.803, *p* < 0.05) and showed a decrease in the alpha frequency band (one-way rm ANOVA; *F*_(1, 7)_ = 1909.767, *p* < 0.05) compared to those of the prestimulus phase. At 1 d, the mean PSD of the stimulus phase decreased in the delta, theta, alpha, and beta bands (one-way rm ANOVA; *F*_(1, 7)_ = 111.719, *p* < 0.05; *F*_(1, 7)_ = 360.460, *p* < 0.05; *F*_(1, 7)_ = 850.932, *p* < 0.05; and *F*_(1, 7)_ = 830.523, *p* < 0.05) compared to those of the prestimulus phase. At 13 d, the mean PSD of the stimulus phase showed a significant decrease in the delta and theta bands (one-way rm ANOVA; *F*_(1, 6)_ = 1243.328, *p* < 0.05 and *F*_(1, 6)_ = 296.415, *p* < 0.05) compared to those of the prestimulus phase. At 15 d, the mean PSD of the delta, theta, alpha, beta, and gamma bands significantly decreased during the stimulus phase (one-way rm ANOVA; *F*_(1, 6)_ = 68.989, *p* < 0.05; *F*_(1, 6)_ = 1015.063, *p* < 0.05; *F*_(1, 6)_ = 191.674, *p* < 0.05; *F*_(1, 6)_ = 2297.207, *p* < 0.05; and *F*_(1, 6)_ = 42.677, *p* < 0.05) compared to those of the prestimulus phase (Figure 3(c)).

### 3.3. After EA, Synchronous Neural Oscillations of Pain Memory-Related Behaviors Were Observed during Nociceptive Behavioral Testing

**Figure 4:**
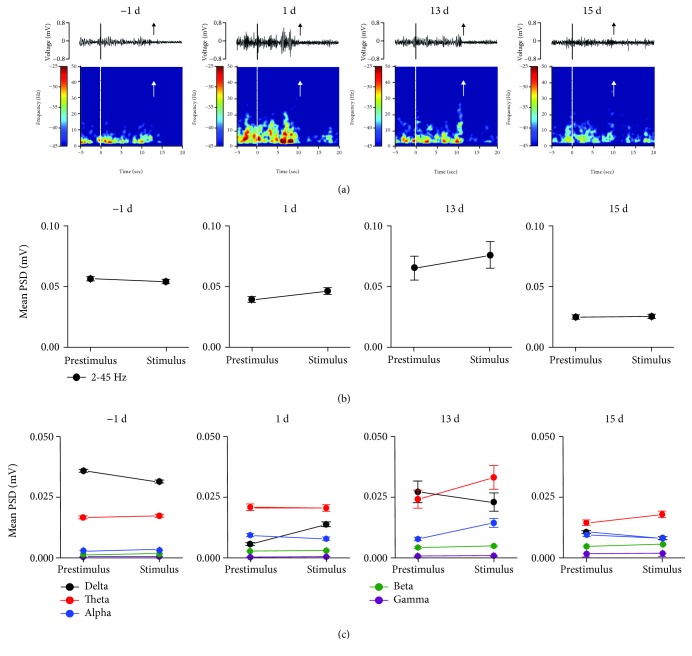
After EA, synchronous LFPs were observed during nociceptive behavioral testing. (a) After EA, a spontaneous recording and real-time spectrogram of LFP signals of the prestimulus, stimulus, and poststimulus phases. (b) After EA, the change in the mean PSD (2-45 Hz) was observed between the prestimulus and stimulus phases in the rACC. (c) After EA, the change in the mean PSD was observed at different frequency bands between the prestimulus and stimulus phases in the rACC. ^∗^*p* < 0.05 compared to the prestimulus phase of the same band. Five frequency band intervals were considered: delta (2–4 Hz), theta (4–9 Hz), alpha (9–15 Hz), beta (15–30 Hz), and gamma (30–45 Hz).

After EA, compared to the prestimulus phase, the early declining amplitude and PSD of the stimulus phase were not observed at 1 d, 13 d, and 15 d (Figure 4(a)).

At -1 d, 1 d, 13 d, and 15 d, there were no significant differences in the mean PSD of the prestimulus and stimulus phases (one-way rm ANOVA; all *p* > 0.05) (Figure 4(b)). Furthermore, Figure 4(c) shows that at -1 d, 13 d, and 15 d, there was no significant difference in the mean PSD of each frequency band between the stimulus and prestimulus phases (one-way rm ANOVA; all *p* > 0.05). At 1 d, the mean PSD in the delta band during the stimulus phase was higher than that during the prestimulus phase (one-way rm ANOVA; *F*_(1, 6)_ = 17.453, *p* < 0.05).

### 3.4. Pain Memory-Related Behaviors Observed during Aversive Behavioral Testing

**Figure 5:**
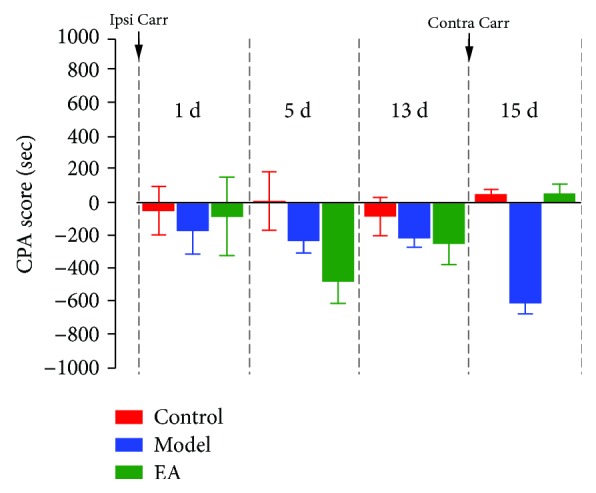
Values of aversive behavioral testing. ^∗^*p* < 0.05 compared to the control group at the same time; ^#^*p* < 0.05 compared to the model group at the same time. *N* = 7. Abbreviation: Ipsi Carr = ipsilateral carrageenan injection, Contra Carr = contralateral carrageenan injection.

Figure 5 shows that there were changes between the control, model, and EA groups at 1 d, 5 d, 13 d, and 15 d (two-way rm ANOVA; groups: *F*_(2, 36)_ = 2.892, *p* > 0.05; time: *F*_(3, 60)_ = 1.066, *p* > 0.05; and groups × time: *F*_(6, 60)_ = 3.079, *p* < 0.05; *n* = 7). The results observed at 15 d show that score values of the model group were lower than those of the control group (*p* < 0.05, Bonferroni test); compared to those of the model group, the score values of the EA group were significantly increased (*p* < 0.05, Bonferroni test). Together, these data suggest that the second injury stimulated the aversive memory in the pain-paired compartments (conditioning phase) of the model rats, spurring significantly aversive avoidance behaviors. EA can inhibit aversive memory induced by a second injury, and the rats exhibited no aversive avoidance behaviors.

### 3.5. Synchronous Neural Oscillations of Pain Memory-Related Behaviors Observed during Aversive Behavioral Testing

**Figure 6:**
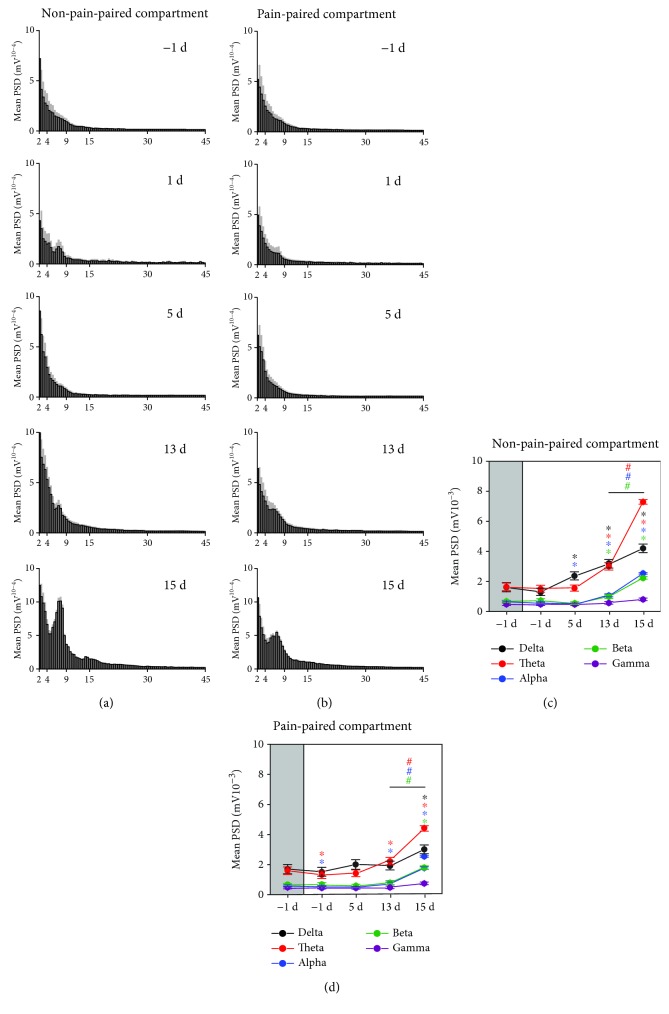
Synchronous LFPs during aversive behavioral testing. (a) Mean PSD histogram of the non-pain-paired compartment (the gray area indicates the standard deviation). (b) Mean PSD histogram of the pain-paired compartment (the gray area indicates the standard deviation). (c) Mean PSD of rACC LFPs for five frequency bands in the non-pain-paired compartment. ^∗^*p* < 0.05 compared to -1 d; ^#^*p* < 0.05 compared to 13 d. (d) Mean PSD of rACC LFPs for five frequency bands in the pain-paired compartment. ^∗^*p* < 0.05 compared to -1 d; ^#^*p* < 0.05 compared to 13 d. Five frequency band intervals were considered: delta (2–4 Hz), theta (4–9 Hz), alpha (9–15 Hz), beta (15–30 Hz), and gamma (30–45 Hz).

Our data indicate that when rats were moving freely in the non-pain-paired compartments, the mean PSD gradually increased, and specifically at 15 d, the mean PSD in the theta band reached a pronounced peak value (Figure 6(a)). When the rats were moving in the pain-paired compartment, the mean PSD also gradually increased, and at 15 d, the mean PSD in the theta band also increased (Figure 6(b)).

Next, we analyzed the mean PSD in five frequency bands. Compared to that observed at -1 d, the mean PSD observed at 1 d in the theta and alpha bands decreased in the pain-paired compartment (one-way rm ANOVA; *F*_(1, 7)_ = 52.347, *p* < 0.05 and *F*_(1, 7)_ = 60.954, *p* < 0.05; Figure 6(d)). The mean PSD at 5 d in the delta band increased but that of the alpha band decreased in the non-pain-paired compartment (one-way rm ANOVA; *F*_(1, 7)_ = 90.105, *p* < 0.05 and *F*_(1, 7)_ = 54.211, *p* < 0.05; Figure 6(c)). The mean PSD at 13 d in the delta, theta, alpha, and beta bands were increased in the non-pain-paired compartment (one-way rm ANOVA; *F*_(1, 7)_ = 754.585, *p* < 0.05; *F*_(1, 7)_ = 656.647, *p* < 0.05; *F*_(1, 7)_ = 178.395, *p* < 0.05; and *F*_(1, 7)_ = 62.780, *p* < 0.05; Figure 6(c)), while it increased in the theta and alpha bands in the pain-paired compartment at the same time (one-way rm ANOVA; *F*_(1, 7)_ = 161.090, *p* < 0.05 and *F*_(1, 7)_ = 56.208, *p* < 0.05; Figure 6(d)). The mean PSD at 15 d in the delta, theta, alpha, and beta bands increased in the non-pain-paired compartment (one-way rm ANOVA; *F*_(1, 6)_ = 40.680, *p* < 0.05; *F*_(1, 6)_ = 371.190, *p* < 0.05; *F*_(1, 6)_ = 444.247, *p* < 0.05; and *F*_(1, 6)_ = 139.694, *p* < 0.05; Figure 6(c)), while in the theta, alpha, and beta bands, it increased in the pain-paired compartment (one-way rm ANOVA; *F*_(1, 6)_ = 83.732, *p* < 0.05; *F*_(1, 6)_ = 124.425, *p* < 0.05; and *F*_(1, 6)_ = 63.617, *p* < 0.05; Figure 6(d)). Note that compared to the mean PSD at 13 d, the PSD of the theta, alpha, and beta bands significantly increased in the two compartments at 15 d (one-way rm ANOVA; non-pain-paired compartment: *F*_(1, 6)_ = 194.174, *p* < 0.05, *F*_(1, 6)_ = 163.629, *p* < 0.05, and *F*_(1, 6)_ = 58.376, *p* < 0.05; pain-paired compartment: *F*_(1, 6)_ = 50.593, *p* < 0.05, *F*_(1, 6)_ = 84.377, *p* < 0.05, and *F*_(1, 6)_ = 41.596, *p* < 0.05; Figures 6(c) and 6(d)).

### 3.6. After EA, Synchronous Neural Oscillations of Pain Memory-Related Behaviors Were Observed during Aversive Behavioral Testing

**Figure 7:**
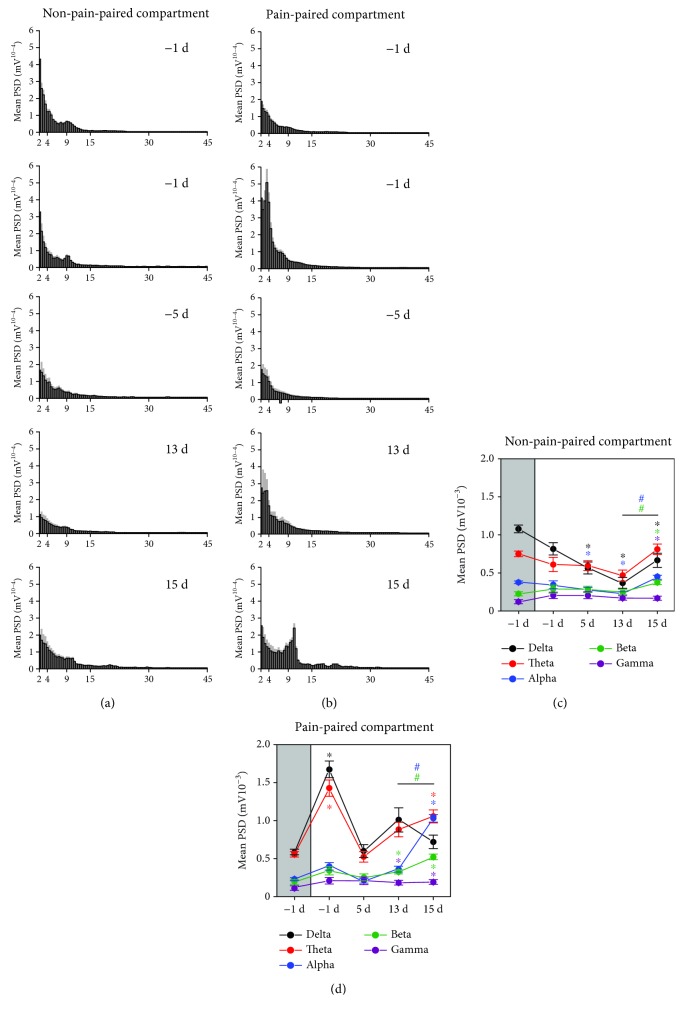
After EA, synchronous LFPs were observed during aversive behavioral testing. (a) After EA, the mean PSD histogram of the non-pain-paired compartment (the gray area indicates the standard deviation). (b) After EA, the mean PSD histogram of the pain-paired compartment (the gray area indicates the standard deviation). (c) After EA, the mean PSD of rACC LFPs for five frequency bands in the non-pain-paired compartment. ^∗^*p* < 0.05 compared to -1 d; ^#^*p* < 0.05 compared to 13 d. (d) After EA, the mean PSD of rACC LFPs for five frequency bands in the pain-paired compartment. ^∗^*p* < 0.05 compared to -1 d; ^#^*p* < 0.05 compared to 13 d. Five frequency band intervals were considered: delta (2–4 Hz), theta (4–9 Hz), alpha (9–15 Hz), beta (15–30 Hz), and gamma (30–45 Hz).

After EA, when the rats were moving freely in the pain-paired compartments at 15 d, the mean PSD in the alpha band reached an obvious peak value rather than in the theta band (Figure 7(b)).

We further analyzed the mean PSD in five frequency bands. Compared to the mean PSD observed at -1 d, the PSD observed at 1 d in the delta and theta bands increased in the pain-paired compartment at 1 d (one-way rm ANOVA; *F*_(1, 7)_ = 156.429, *p* < 0.05 and *F*_(1, 7)_ = 88.682, *p* < 0.05; Figure 7(d)). The PSD at 5 d in the delta and alpha bands decreased in the non-pain-paired compartment (one-way rm ANOVA; *F*_(1, 7)_ = 121.593, *p* < 0.05 and *F*_(1, 7)_ = 30.721, *p* < 0.05; Figure 7(c)). The PSD at 13 d in the delta and alpha bands decreased in the non-pain-paired compartment (one-way rm ANOVA; *F*_(1, 7)_ = 95.330, *p* < 0.05 and *F*_(1, 7)_ = 31.672, *p* < 0.05; Figure 7(c)), while in the beta and gamma bands, it increased in the pain-paired compartment (one-way rm ANOVA; *F*_(1, 7)_ = 39.571, *p* < 0.05 and *F*_(1, 7)_ = 67.012, *p* < 0.05; Figure 7(d)). The PSD at 15 d in the delta, beta, and gamma bands increased in the non-pain-paired compartment (one-way rm ANOVA; *F*_(1, 7)_ = 57.185, *p* < 0.05; *F*_(1, 7)_ = 222.434, *p* < 0.05; and *F*_(1, 7)_ = 36.979, *p* < 0.05; Figure 7(c)), while in the theta, alpha, beta, and gamma bands, it increased in the pain-paired compartment (one-way rm ANOVA; *F*_(1, 7)_ = 95.254, *p* < 0.05; *F*_(1, 7)_ = 444.281, *p* < 0.05; *F*_(1, 7)_ = 408.482, *p* < 0.05; and *F*_(1, 7)_ = 95.737, *p* < 0.05; Figure 7(d)). Note that compared to the mean PSD observed at 13 d, the PSD of the alpha and beta bands significantly increased in the two compartments at 15 d (one-way rm ANOVA; *F*_(1, 7)_ = 127.105, *p* < 0.05 and *F*_(1, 7)_ = 54.631, *p* < 0.05; Figures 7(c) and 7(d)) rather than in the theta band.

## 4. Discussion

The results showed that EA plays an important role in regulating pain memory-related behavior and synchronous neural oscillations in the rACC. During pain memory retrieval, a nonpain stimulus generates a neural oscillatory reaction similar to the pain stimulus in the rACC. This may be a mechanism of the decrease in PWTs induced by pain memory. After EA, the nonpain stimulus did not induce decreased neural oscillatory activity in advance in the rACC. It may be that EA suppresses pain memory in nociceptive behavior. In addition, we observed an extensive change in neural oscillations in rats with aversive behavior, including power enhancement in the theta, alpha, and beta bands. EA only inhibited theta power in the rACC, and alpha and beta power levels still increased. These results indicate that EA, through the downregulation of theta power, inhibited the retrieval of aversive memory and regulated pain memory-induced aversive behavior.

### 4.1. The Paw Stimulus-Induced Changes in Neural Oscillations in the rACC

Our data show that the PWTs of the model group were significantly decreased compared to the control group at 15 d. This means that the pain memory in the model group was retrieved after the injury of the contralateral hind paws. It has previously been reported that a pain stimulus can rapidly inhibit spontaneous neural oscillations that can open the gates of sensory and motor systems, and a predictive warning of pain can be used to prepare for subsequent individual processing and for responses to external stimuli [37]. However, according to our results, a nonpain stimulus can also inhibit spontaneous neural oscillations. At 15 d, neural oscillations in the rACC were inhibited during tactile and pressure sensation. Then, the gate of the motor system opened in advance, and a paw withdraw response occurred.

Remarkably, neural oscillations declined early, but not for PWTs at 13 d. However, neural oscillations were inhibited early on, and PWTs also declined at 15 d. This suggests that at 13 d, the rats were hypersensitive to the nonpain stimulus, but they only paid attention to the nonpain stimulus rather than exhibiting paw withdrawal behaviors to escape. At 15 d, the rats were more sensitive after the second injury. Although the primary tissue injury to the left hind paw has recovered at 15 d, an early warning and attention can still be dedicated to the nonpain stimulus to withdraw paws early to avoid potential damage. This suggests that the paw withdrawal reaction induced by acute injury may be an escape reaction occurring through the transmission of peripheral sensory information to the central system (bottom-up), while the paw withdrawal reaction induced by pain memory may serve as a predictive warning and a protective reaction through the transmission of the central cognitive information to the periphery (top-down).

### 4.2. After EA, the Paw Stimulus Induced Changes in Neural Oscillations in the rACC

The PWTs of the EA group were significantly increased compared to those of the model group at 15 d. This indicates that EA suppresses the pain memory retrieval of left paws.

In addition, at 1 d, the PWTs of the EA group were enhanced relative to those of the model group, which means that EA likely recovers the acute pain threshold by modulating the rACC. However, the early declining PSD of rACC was not observed during the stimulus period. This indicates that EA may suppress the rACC prereaction to the nonpain stimulus during the acute period. At 13 d and 15 d, the paw nonnoxious stimulus did not induce the early decline of neural oscillations in the rACC. This could partly explain why we did not observe hyperpathia induced by pain memory. This also shows that EA may inhibit the oversensitive protective response induced by pain memory by regulating early warning information processing in the rACC.

In addition, the application of EA started 4 h after carrageenan injection and it ended at 5 d. Although the application of EA was not continued, the pain memory did not occur in the EA group after the second injury. Therefore, EA not only plays an acute analgesic role but also eliminates the subsequent long-term overprotection effect induced by pain memory.

### 4.3. Aversive Stimuli Induced Changes in Neural Oscillations in the rACC

CPA scores represent the time spent in the pain-paired compartment in the test session minus the time spent in the same compartment in the preconditioning (baseline) session. The CPA score obtained in this way is the difference between each rat before and after, and it can better show the changes before and after itself. Therefore, the CPA score shows the changes in the degree of aversion to the pain-paired compartment over time. Score values of the pain-paired compartment show that pain-related aversive behaviors did not appear after primary paw injury but appeared after the second paw injury, showing that pain-related aversive memory caused the rats to escape from the pain-paired compartments.

Many studies show that the increased theta band is related to memory loss and that the decreased theta band denotes memory arousal [38–40]. In our study, the PSD of the theta band gradually increased over time in pain and non-pain-paired compartments after primary paw injury. These results suggest that the memory for two compartments fades after a conditioning phase at 4 h. Note that the memory of the non-pain-paired compartment fades more quickly than that of the pain-paired compartment, especially at 15 d. Therefore, the PSD of the theta band may be a key mechanism of pain-related aversive memory processing.

### 4.4. After EA, an Aversive Stimulus Induced Changes in Neural Oscillations in the rACC

Rats of the EA group did not exhibit aversive escape reactions after a second paw injury. This shows that EA may suppress aversive memory retrieval by intervening with rACC. However, how EA intervenes with the neural oscillation activity in the rACC is in need of further observation.

Studies have shown that the alpha and beta bands participate in the control of top-down cognitive processes [41], especially alpha bands which are involved in internally directed cognitive processes [42–44]. Our results show that after EA, the PSD at day 15 show no changes in the theta band but show a significant increase in the alpha and beta bands after the second paw injury. Taken together, EA could not only inhibit aversive memory retrieval by intervening in the theta power but could also strengthen the inhibition of top-down control by enhancing the alpha and beta power levels. These two processes work together to suppress aversive memory induced by pain memory.

## 5. Conclusions

Our research, in relation to pain memory, reveals the possible intervening mechanisms of EA in long-term pain. In this study, we observe the effects of EA on pain memory-induced nociceptive and aversive behaviors and on synchronous neural oscillations in the rACC. During nociceptive behavioral testing, a pain memory-induced nonpain stimulus spurred a neural oscillatory reaction in the rACC similar that of a pain stimulus. After EA, the nonpain stimulus did not induce decreased neural oscillatory activity in the rACC until a pain stimulus was applied. During aversive behavioral testing, EA, through the downregulation of theta power, inhibited the retrieval of aversive memory and relieved pain memory-induced aversive behaviors. These results extend previous studies on pain memory and EA. These changes of oscillatory activity may be the hallmarks of EA therapy for pain memory.

## Data Availability

The data used to support the findings of this study are available from the corresponding authors upon request.
